# Multi-laboratory performance assessment of diffuse optics instruments: the BitMap exercise

**DOI:** 10.1117/1.JBO.27.7.074716

**Published:** 2022-06-15

**Authors:** Pranav Lanka, Lin Yang, David Orive-Miguel, Joshua Deepak Veesa, Susanna Tagliabue, Aleh Sudakou, Saeed Samaei, Mario Forcione, Zuzana Kovacsova, Anurag Behera, Thomas Gladytz, Dirk Grosenick, Lionel Hervé, Turgut Durduran, Karolina Bejm, Magdalena Morawiec, Michał Kacprzak, Piotr Sawosz, Anna Gerega, Adam Liebert, Antonio Belli, Ilias Tachtsidis, Frédéric Lange, Gemma Bale, Luca Baratelli, Sylvain Gioux, Kalyanov Alexander, Martin Wolf, Sanathana Konugolu Venkata Sekar, Marta Zanoletti, Ileana Pirovano, Michele Lacerenza, Lina Qiu, Edoardo Ferocino, Giulia Maffeis, Caterina Amendola, Lorenzo Colombo, Lorenzo Frabasile, Pietro Levoni, Mauro Buttafava, Marco Renna, Laura Di Sieno, Rebecca Re, Andrea Farina, Lorenzo Spinelli, Alberto Dalla Mora, Davide Contini, Paola Taroni, Alberto Tosi, Alessandro Torricelli, Hamid Dehghani, Heidrun Wabnitz, Antonio Pifferi

**Affiliations:** a Politecnico di Milano, Dipartimento di Fisica, Milano, Italy; b Physikalisch-Technische Bundesanstalt (PTB), Berlin, Germany; c Université Grenoble Alpes, CEA, LETI, DTBS, Grenoble, France; d University of Birmingham, School of Computer Science, Birmingham, United Kingdom; e The Institute of Photonic Sciences (ICFO), Castelldefels, Spain; f Nalecz Institute of Biocybernetics and Biomedical Engineering, Warsaw, Poland; g University Hospitals Birmingham, National Institute for Health Research Surgical Reconstruction and Microbiology Research Centre, Birmingham, United Kingdom; h UCL, Department of Medical Physics & Biomedical Engineering, London, United Kingdom; i University of Cambridge, Department of Engineering and Department of Physics, Cambridge, United Kingdom; j University of Strasbourg, ICube Laboratory, Strasbourg, France; k University Hospital Zurich, Biomedical Optics Research Laboratory, Department of Neonatology, Zurich, Switzerland; l IPIC, Tyndall National Institute, Cork, Ireland; m South China Normal University, School of Software, Guangzhou, China; n Istituto di Fotonica e Nanotecnologie, Milano, Italy; o Politecnico di Milano, Dipartimento di Elettronica, Informazione e Bioingegneria, Milano, Italy

**Keywords:** diffuse optics, near-infrared spectroscopy, absorption, scattering, phantom

## Abstract

**Significance:**

Multi-laboratory initiatives are essential in performance assessment and standardization—crucial for bringing biophotonics to mature clinical use—to establish protocols and develop reference tissue phantoms that all will allow universal instrument comparison.

**Aim:**

The largest multi-laboratory comparison of performance assessment in near-infrared diffuse optics is presented, involving 28 instruments and 12 institutions on a total of eight experiments based on three consolidated protocols (BIP, MEDPHOT, and NEUROPT) as implemented on three kits of tissue phantoms. A total of 20 synthetic indicators were extracted from the dataset, some of them defined here anew.

**Approach:**

The exercise stems from the Innovative Training Network BitMap funded by the European Commission and expanded to include other European laboratories. A large variety of diffuse optics instruments were considered, based on different approaches (time domain/frequency domain/continuous wave), at various stages of maturity and designed for different applications (e.g., oximetry, spectroscopy, and imaging).

**Results:**

This study highlights a substantial difference in hardware performances (e.g., nine decades in responsivity, four decades in dark count rate, and one decade in temporal resolution). Agreement in the estimates of homogeneous optical properties was within 12% of the median value for half of the systems, with a temporal stability of <5% over 1 h, and day-to-day reproducibility of <3%. Other tests encompassed linearity, crosstalk, uncertainty, and detection of optical inhomogeneities.

**Conclusions:**

This extensive multi-laboratory exercise provides a detailed assessment of near-infrared Diffuse optical instruments and can be used for reference grading. The dataset—available soon in an open data repository—can be evaluated in multiple ways, for instance, to compare different analysis tools or study the impact of hardware implementations.

## 1 Introduction

Diffuse optics (DO) encompasses a range of photonics tools based on the study of random photon migration in highly scattering media—biological tissues in particular. Due to its unique features, DO is emerging as a powerful means for clinical or homecare diagnostics.^1^ The basic physics of DO is related to the detection of temporal or spatial alteration in photon distribution re-emitted on the tissue surface.^2^ Due to the low power (typically few mW) of injected near-infrared light (600- to 1100-nm range), DO is inherently noninvasive. The photon temporal (or spatial) distribution carries information on the absorption—ultimately related to tissue chemical composition, such as water, lipid, collagen content, oxy- and deoxy-hemoglobin concentration, cytochrome c-oxidase^3^—and scattering properties—linked to tissue microstructure. Further, DO is one of the few noninvasive modalities capable of providing functional information, e.g., brain or muscle activation.^4^ It can be operated noncontact through remote light illumination and collection.^5^ It explores the tissue well below the skin at depths of up to 2 to 4 cm. It can provide quantitative operator-independent assessment of the tissue status, such as hemoglobin oxygenation level in the brain. Finally, it is highly scalable, sharing the same technology from large clinical tomographic systems down to wearable devices or homecare appliances.^6^ For all these aspects, DO is attracting more and more interest in many fields, such as monitoring vital signs like brain oxygenation in critical care or during interventions,^7^ tumor diagnostics as for breast cancer,^8^ investigating the impact of lifestyle and nutrition on our body,^9^ or other fields such as neuroscience and psychology,^10^ sports, and leisure.^11^ Even further, the operator independence, the depth sensitivity, and the scalability in addition to noninvasiveness make this option attractive for telemedicine and homecare of patients remotely.

Performance assessment and standardization (PAS) is needed to secure solid growth in the field of DO and in general of biophotonics tools for clinical diagnostics. By PAS, we mean all steps providing an objective quantitative assessment of some key figures-of-merit (FOMs) of a given device related to its clinical use. The first reason for the adoption of PAS procedures is the need to anticipate possible technical problems from the clinical ward back to the laboratory bench. Indeed, many issues or poor performances hampering clinical studies could be identified much earlier with great savings in efforts and public spending and fewer ethical concerns. PAS is useful to benchmark development and upgrades so to drive new designs or improvements. PAS improves the reliability and comparability of clinical studies by setting a common ground for the comparison of instruments. Also, it facilitates machine learning algorithms by providing a testing dataset related to some universal features. Open data and open science can benefit from a common ground of PAS since this improves interoperability of data and comparison of different datasets. Industrial deployment is advantaged since PAS FOMs can be translated more easily in technical specification and can be also the basis for industrial standards, which is the ultimate step of PAS. Finally, also patients and the healthcare system benefit from PAS by improving the technical quality of instrument and reduce health-related costs by increasing reliability. Industrial standards and procedures for clinical validation are already well-rooted in the DO community. Recently, much awareness of the need for PAS for biophotonics and related tools has been raised by the scientific community,^12^^–^^14^ scientific publishers,^15^^,^^16^ funding, and regulatory bodies.^17^ Our goal is to anticipate many issues in the early stages and support the culture of PAS in the whole process.

Our work within the BitMap exercise capitalizes on over two decades of joint efforts within the DO field. In reviewing previous works, we will focus only on multi-laboratory actions, for the sake of brevity on one side but also for a methodological reason since PAS necessarily requires consensus from many players to be effective. The three pillars of the BitMap exercise are three protocols for PA of DO instruments which were elaborated in the framework of large European projects or network consortia, namely the basic instrument performance (BIP),^18^ MEDPHOT,^19^ and NEUROPT^20^ protocols, involving 7 to 10 different institutions each. These codify the key FOMs, procedures and phantoms for testing a DO instrument from the side of (i) the BIP; (ii) the capability to retrieve the optical properties—absorption ($\mu_{a}$) and reduced scattering coefficient ($\mu_{s}^{\prime }$)—of a homogeneous turbid medium (MEDPHOT); (iii) the detection, localization, and quantification of optical inhomogeneities buried into a diffusive medium (NEUROPT). While these protocols were proposed for specific classes of DO instruments—e.g., BIP for time-domain single-photon counting systems, MEDPHOT for assessment of tissue properties as in breast spectroscopy, nEUROPt for time-domain functional brain imagers—yet their scope can be quite general, as stated by the large variety of techniques and applications covered by the tested BitMap instruments as reported in Sec. 3.3.

Another key multi-laboratory undertaking is the accurate characterization of tissue-equivalent phantoms to be used to test the systems in realistic scenarios. A multi-laboratory exercise,^21^ involving eight institutions, led to an accurate characterization (with uncertainty within 2%) of the intrinsic absorption coefficient of India ink and the intrinsic reduced scattering coefficient of Intralipid-20%, which can now be used as an easily reproducible reference materials for liquid phantoms. At a different stage, our work was inspired also by multi-laboratory comparison of instruments enrolled in multicentric clinical studies. The ACRIN 6991 initiative^22^ involving six centers provided an extensive test on equivalent phantoms of instruments engaged in monitoring and predicting neoadjuvant chemotherapy treatment for breast cancer. The aim of the SafeBoosC international randomized phase III^23^ clinical trial^24^ is to determine the benefit of cerebral oximeters in preventing brain lesions in preterm infants. Since currently, cerebral oximeters provide systematically different values of tissue oxygen saturation,^25^ the different oximeters were compared in phantoms^26^ before being eligible for the trial to achieve comparability of the alarm limits.

The BitMap exercise presented in this paper is the largest multi-laboratory comparison of DO instruments, encompassing 12 institutions, and 28 systems. It is an integrated initiative with three separate actions—as detailed in Sec. 2—that are “collection of experimental data” (Action1), “consolidation of open data” (Action2), and “common analysis of open data” (Action3). The key aim is to enforce the culture of PAS in the DO community and beyond and propose a common methodology that could be adopted in other environments. Further, we compare the performance of the instruments based on various data acquisition techniques and analysis methods. Finally, the work is aimed to set a reference picture of DO instrument performances to grade instrument upgrades and new developments and to provide figures in design and simulation studies.

The scope is restricted to DO instruments based on $\mu_{a}$ and $\mu_{s}^{\prime }$ or directly related parameters (e.g., light attenuation) as key measurable. It includes different approaches (e.g., time-resolved, frequency-domain, continuous-wave multidistance, spatial frequency domain as well as different application fields (e.g., optical mammography, brain imaging, tissue spectroscopy). We exclude sources of optical contrast other than $\mu_{a}$ or $\mu_{s}^{\prime }$ such as fluorescence or speckle.

The paper is structured as follows: first, we present the BitMap exercise in the context of PAS (Sec. 2), then we describe the protocols, phantoms, instruments, and analysis tools adopted in the exercise (Sec. 3), next we showcase exemplary results explaining the meaning of each individual test and propose a set of 20 synthetic indicators (Sec. 4), further we sum-up all performance indicators in a summary table and discuss needs and perspectives highlighted by this study (Sec. 5), finally, we draw the conclusions and the key messages of this study (Sec. 6).

## 2 Methodology of the BitMap Exercise

**Fig. 1:**
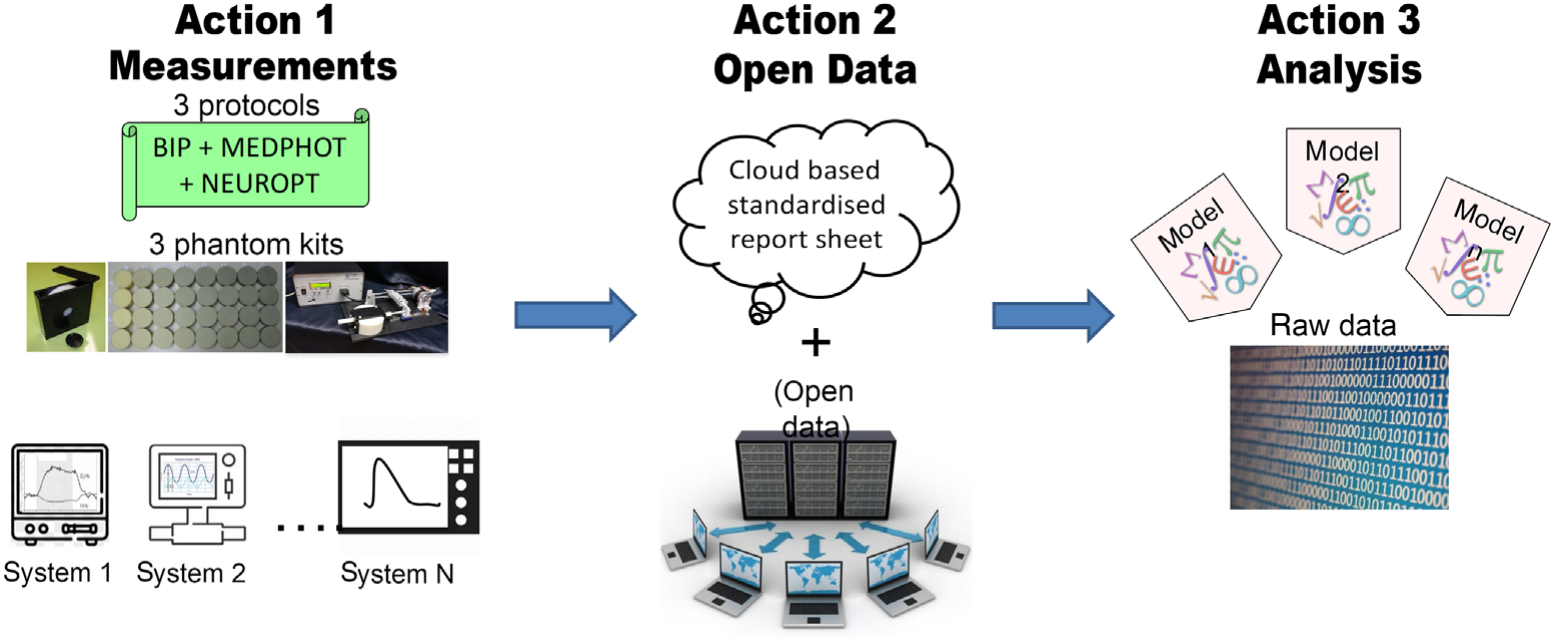
The three actions involved in the BitMap exercise.

The BitMap exercise originated from the Marie Skłodowska–Curie Innovative Training Network “Brain injury and trauma monitoring using advanced photonics” (BitMap) funded by the European Commission within the Horizon 2020 program and then evolved to include other researchers all over Europe. The whole initiative is divided into three actions, as depicted in Fig. 1.

Action1 deals with the gathering of experimental data. The instruments were challenged on three internationally agreed protocols (BIP, MEDPHOT, NEUROPT, see Sec. 3) implemented with three phantom kits (responsivity phantom, MEDPHOT kit, switchable phantom, see Sec. 3). The three phantom kits and sets of instructions were circulated among laboratories over a period of about 2 years and an experienced researcher joined the local teams in most cases to grant uniform execution and quality control. At this stage, the data were processed by the local researchers adopting their own tools so to capture performances under the routine operation of the devices. The idea behind this is not to identify the best instrument performances achievable with the device, but rather to capture the real performances expected in a clinical scenario.

All data will be made available as open data in Action2 adopting the new SNIRF^27^ format proposed by the Society for Functional Near-Infrared Spectroscopy. This will permit reuse and further exploitation of the data. In this first paper, due to the vastity of results, we opted to provide only a descriptive picture of the outcome, just with a few examples for clarifications. A more insightful analysis of the correlation of specific hardware features with results could be pursued in focused works, also by other groups.

In particular, in Action3 all data will be processed using shared analysis tools so to disentangle variability due to the operator or the analysis method. Also, it will be ground to test differences among various analysis tools.

An excerpt of the results of Action1 is presented in this paper, while the open data set is annotated in a companion paper in progress. The outcome of Action3 is still ongoing and will be presented later.

## 3 Materials and Methods

In this section, we briefly discuss the protocols and phantoms used in the BitMap exercise and all the instruments involved.

### 3.1 Protocols

Table 1 summarizes the content of the three protocols for PAS of DO-based instrumentation mentioned above, namely the BIP, MEDPHOT, and NEUROPT. Each of these protocols is further divided into individual tests. A more detailed description of these tests will be presented in the results section. The measurands considered for the assessment of the instruments were limited to those relying on the estimate of homogeneous optical properties ($\mu_{a}$, $\mu_{s}^{\prime }$) and the contrast measured on the inhomogeneous sample.

**Table 1:** Summary of the protocols, phantoms, and selected tests used for the BitMap exercise.

Protocol	Tests	Phantoms	Measurable	Purpose
BIP	• IRF	Responsivity solid phantom	• IRF(profile, background, stability)	Characterize the basic instrumental performances
	• DNL			
	• Responsivity			
			• DNL	
			• Responsivity	
MEDPHOT	• Accuracy	Matrix of 32 homogeneous phantoms	• Absorption ($\mu_{a}$)	Characterize the ability of the instrument to accurately recover homogeneous optical properties
	• Linearity		• Reduced scattering ($\mu_{s}^{\prime }$)	
	• Uncertainty			
	• Stability			
	• Reproducibility			
NEUROPT	• Detection	Solid switchable phantom	• Contrast	Characterize the ability of the instrument to detect an inhomogeneity
	• Localization		• CNR	
	• Quantification			

IRF, instrument response function; DNL, differential nonlinearity; CNR, contrast-to-noise ratio.

### 3.2 Phantoms

**Fig. 2:**
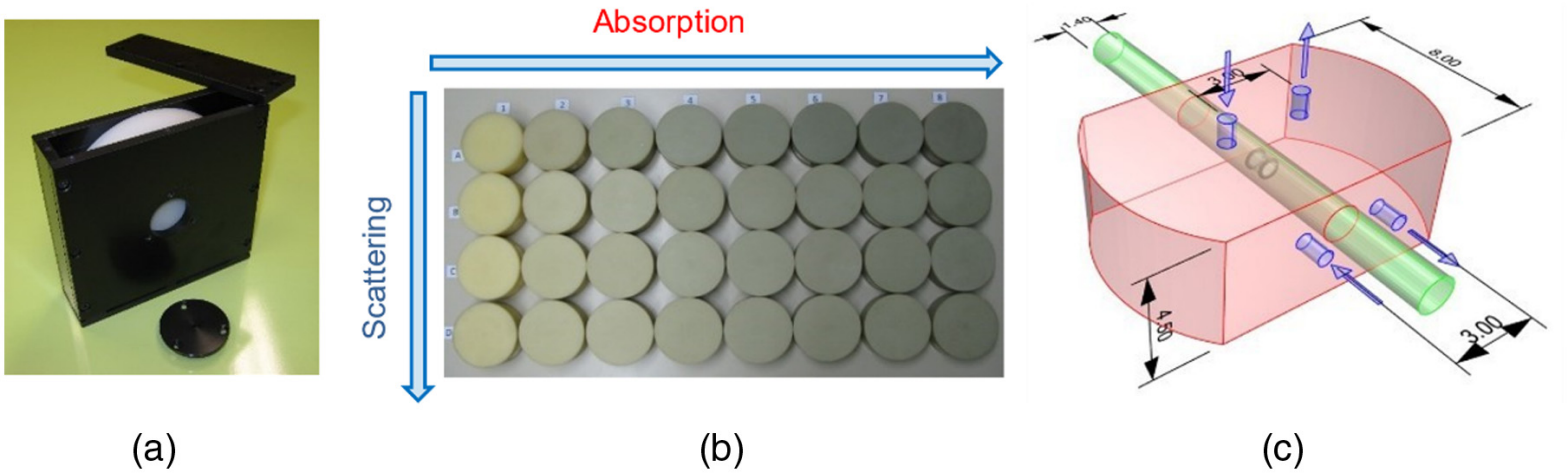
All the phantoms used for this exercise. (a) Responsivity phantom, (b) MEDPHOT kit, and (c) the solid switchable phantom (dimensions in cm).

Three sets of phantoms linked to each of the above-mentioned protocols and thoroughly characterized in previous multi-laboratory studies were chosen for this exercise. In particular, we opted for solid phantoms to facilitate reproducibility of results and easy application of the tests. The phantoms were circulated sequentially to all laboratories following a round-robin scheme. In detail, for the specific test of the BIP protocol, we chose a responsivity phantom^18^ [Fig. 2(a)] which is a solid homogeneous turbid slab of 2-cm thickness and 10.5-cm diameter with accurately characterized diffuse transmittance factor used to create a defined diffuse light source to evaluate the overall responsivity of the detection part of the instrument. For the MEDPHOT protocol [Fig. 2(b)], we adopted the MEDPHOT kit which is a set of 32 homogeneous solid phantoms spanning a wide range of absorption and reduced scattering properties.^19^ At the time of fabrication, >20 years ago, the nominal properties at 800 nm calculated from the concentrations of black toner and ${\mathrm{TiO}}_{2}$ powder were assumed to be: $\mu_{a}$ from 0 to $0.35\;{\mathrm{cm}}^{-1}$ in steps of $0.05\;{\mathrm{cm}}^{-1}$, and $\mu_{s}^{\prime }$ from 5 to $20\;{\mathrm{cm}}^{-1}$ in steps of $5\;{\mathrm{cm}}^{-1}$. Finally, for the nEUROPt protocol [Fig. 2(c)] we used a solid switchable phantom^28^ that is a solid epoxy resin matrix ($120\times 80\times 45\;{\mathrm{mm}}^{3}$) with standard optical properties ($\mu_{a}=0.1\;{\mathrm{cm}}^{-1}$ and $\mu_{s}^{\prime }=10\;{\mathrm{cm}}^{-1}$ at 700 nm) holding a rod which can slide along a direction parallel to the upper surface and set at a depth of 1.5 cm. The rod embeds a black cylinder (length 0.5 cm, diameter 0.5 cm) which provides an optical perturbation equivalent to an absorption change of $0.17\;{\mathrm{cm}}^{-1}$ assuming $\mu_{s}^{\prime }=10\;{\mathrm{cm}}^{-1}$ for the background.^29^

### 3.3 Instruments and Institutions

A total of 28 instruments were enrolled for this PA exercise. These instruments are listed in Table 2 along with some basic information on the modality and application. To give the reader an unbiased picture of the study a unique enrollment ID for each instrument will be used to represent the instrument from this point on. Not all the tests mentioned above are applicable to all the instrumentation presented in this table. For instance, the continuous wave (CW)-only instruments (ID #4, #9 and #20) were not assessed using the BIP protocols, which are meant for time-domain (TD) instrumentation, nor with MEDPHOT, which requires the estimation of the optical properties, which was not feasible for the above-mentioned systems. In other cases, the mechanical design or other similar obstacles restrict the application of certain tests or protocols to certain instruments, as in the case of ID #21 and #27 which are designed to work in transmittance alone whereas the nEUROPt protocol requires a reflectance geometry. Similarly, the design of instrument #7 precludes the power measurement of the source (at a particular wavelength) thus making the instrument invalid for the Responsivity measurement of the BIP protocol. Table 3 provides a short overview of the different tests performed for each individual instrument. Irrespective of these limitations the cohort of instruments challenged under every test is still large enough to provide a valuable dataset for the other two actions. Another dimension in which the instruments enrolled show good variability is the technology readiness level (TRL).^62^ A numeric scale from TRL1 to TRL9 stages the maturity of the technology, where TRL1 stands for *basic principles observed* and TRL9 to *final deployment in an operational environment*. System #2, e.g., is based on an emerging technology involving a large area silicon photomultiplier (SiPM) detector and hence is low on the TRL scale. On the other hand, other instruments enrolled in clinical studies rank relatively higher. Finally, the ISS, NIRO,^33^^,^^34^ and Artinis instruments (#4, #5, and 20) are commercially manufactured instruments routinely used in a bedside clinical environment, thus exhibiting the highest possible TRL.

**Table 2:** List of instruments involved in the BitMap exercise.

Instrument name	Institute	ID	Modality	Application	Analysis	TRL	Date	Ref
Clinical broadband TD-DOS	POLIMI^a^	1	TD	Spectroscopy	DE	5	02-2019	30
TD large area SiPM system	POLIMI^a^	2	TD	Oximetry	DE	3	02-2019	31
TD lab system with HPM	PTB^b^	3	TD	Spectroscopy	MC	4	03-2019	32
NIRO 200NX	UHB/UoB^c^	4	CW	Oximetry	SRS	8	03-2019	33
ISS OXIPLEX-TS	UHB/UoB^c^	5	FD	Oximetry	FDMD	8	02-2019	34
TD multiwavelength system	IBIB^d^	7	TD	Spectroscopy	MM	5	03-2019	35,36
TD-DCS laboratory system	IBIB^d^	8	TD	Blood flow	MM	4	02-2019	37
(CYRIL) SRS-CW system	UCL^e^	9	CW	Spectroscopy	SRS	6	02-2019	38
TRS-DCS FLOWer	ICFO^f^	10	TD	Oximetry	DE	7	02-2019	
TD lab system with MCP	PTB^b^	11	TD	Spectroscopy	MC	4	02-2019	39
Clinical TD oximeter	IBIB^d^	13	TD	Oximetry	MM	6	02-2019	40,41
TD optical brain imager	IBIB^d^	14	TD	Oximetry	MM	6	02-2019	42,43
TD MAESTROS	UCL^e^	15	TD	Spectroscopy	DE	4	12-2018	44
LUCA device	POLIMI^a^	16	TD	Spectroscopy	DE	6	02-2019	45
Oximin	IFN-CNR^I^	17	TD	Oximetry	DE	6	03-2019	46
Clinical multichannel oximeter	IFN-CNR^i^	18	TD	Oximetry	DE	6	02-2019	47
Wearable fNIRS (NIRSBOX)	POLIMI^a^	19	TD	Oximetry	DE	6	Jul-2019	48
OctaMon, Artinis	POLIMI^a^	20	CW	Oximetry	DE	8	01-2019	49
Mammot	POLIMI^a^	21	TD	Mammography	DE	6	05-2019	50,51
“Fruit” spectrometer	IFN-CNR^i^	22	TD	Spectroscopy	DE	4	05-2019	52
OCTOPUS	POLIMI^a^	23	TD	Imaging	DE	4	02-2019	53,54
Clinical DCS—BabyLux	POLIMI^a^/ICFO^f^	24	TD	Oximetry	DE	6	02-2019	55
Laboratory broadband TD-DOS	POLIMI^a^	25	TD	Spectroscopy	DE	6	04-2019	56
Laboratory TD-DCS	POLIMI^a^	26	TD	Blood Flow	DE	4	01-2019	57
Mammot v2	POLIMI^a^	27	TD	Mammography	DE	6	11-2019	58
Benchtop DOS	UoS^g^	28	TD	Spectroscopy	DE	4	11-2019	
Multispectral SFDI	UoS^g^	29	SFDI	Imaging	MC	4	07-2020	59,60
NIROT “Pioneer” imager	UoZ^h^	30	FD	Imaging		8	02-2019	61

HPM, hybrid photomultiplier; MCP-PMT, microchannel plate photomultiplier; TD, time domain; CW, continuous wave; FD, frequency domain; SFDI, spatial frequency domain imaging; SRS, spatially resolved spectroscopy; DCS, diffuse correlation spectroscopy; DE, diffusion equation; MC, Monte Carlo; MM, method of moments; DOS, diffuse optical spectroscopy; SiPM, silicon photomultiplier; FDMD, frequency-domain multiple-distance.

ID # 6 and 12 correspond to instruments omitted from the exercise.

a Politecnico di Milano.

b Physikalisch-Technische Bundesanstalt, Berlin.

c University Hospitals Birmingham, Birmingham/ University of Birmingham, Birmingham.

d Nalecz Institute of Biocybernetics and Biomedical Engineering, Warsaw.

e University College London, London.

f The Institute of Photonic Sciences, Barcelona.

g ICube Laboratory, University of Strasbourg, Strasbourg.

h Biomedical Optics Research Laboratory, University Hospital Zurich, Zurich.

i Istituto di Fotonica e Nanotecnologie-CNR, Milan.

**Table 3:** An overview of the different tests applied to each of the instruments enrolled (Y, Yes; N, No).

ID and modality		BIP				MEDPHOT					NEUROPT
		IRF	Resp	DNL	Dark	Lin	Acc	Stab	Noise	Rep	Detection
1	TD	Y	Y	Y	Y	Y	Y	Y	Y	Y	Y
2	TD	Y	Y	Y	Y	Y	Y	Y	Y	Y	Y
3	TD	Y	Y	Y	Y	Y	Y	Y	Y	Y	Y
4	CW	N	N	N	N	N	N	N	N	N	Y
5	FD	N	N	N	N	Y	Y	Y	Y	Y	Y
7	TD	Y	N	Y	Y	Y	Y	Y	Y	Y	Y
8	TD	Y	Y	Y	Y	Y	Y	Y	Y	Y	Y
9	CW	N	N	N	N	N	N	N	N	N	Y
10	TD	Y	N	N	N	Y	Y	Y	Y	N	N
11	TD	Y	N	Y	Y	Y	Y	Y	Y	Y	Y
13	TD	Y	Y	Y	Y	Y	Y	Y	Y	Y	Y
14	TD	Y	Y	Y	Y	Y	Y	Y	Y	Y	Y
15	TD	Y	Y	Y	Y	Y	Y	Y	Y	Y	Y
16	TD	Y	Y	Y	Y	Y	Y	Y	Y	Y	Y
17	TD	Y	Y	Y	Y	Y	Y	Y	Y	Y	Y
18	TD	Y	Y	Y	Y	Y	Y	Y	Y	Y	Y
19	TD	Y	Y	Y	Y	Y	Y	Y	Y	Y	Y
20	CW	N	N	N	N	N	N	N	N	N	Y
21	TD	Y	Y	Y	Y	Y	Y	Y	Y	Y	N
22	TD	Y	Y	Y	Y	Y	Y	Y	Y	Y	Y
23	TD	Y	Y	Y	Y	Y	Y	Y	Y	Y	Y
24	TD	Y	Y	Y	Y	Y	Y	Y	Y	Y	N
25	TD	Y	Y	Y	Y	Y	Y	Y	Y	Y	Y
26	TD	Y	Y	Y	Y	Y	Y	Y	Y	Y	N
27	TD	Y	Y	Y	Y	Y	Y	Y	Y	Y	N
28	TD	Y	Y	Y	Y	Y	Y	Y	Y	Y	N
29	SFDI	N	N	N	N	Y	Y	Y	N	Y	N
30	FD	N	N	N	N	Y	Y	Y	Y	Y	N

### 3.4 Data Analysis

For the Action1 of the exercise, the analysis of the data obtained by each of the instruments was performed individually by the respective institutions using analysis procedures generally used when the corresponding instrument is employed, e.g., in a clinical study. Particularly, for the TD instrumentation, most instruments employed analysis models based on the diffusion equation (diffusion approximation of the radiative transport equation), while some others used the stochastic Monte Carlo (MC)-based models. Further information regarding data analysis for the individual instruments can be found in the instrument references in Table 2.

## 4 Results

The size of the dataset limits the display of the results of individual tests for all the instruments enrolled in the exercise. Rather, results are condensed to a single (or at most two) numeric values for each test, the aforementioned FOM. Exemplary plots with results from a few instruments are also plotted for specific tests in order to facilitate the readers’ understanding.

### 4.1 Basic Instrument Performance

As mentioned, this protocol concerns primarily the TD instrumentation and more specifically deals with recording the basic characteristics which influence the quality and accuracy of measurements in clinical applications. The basic instrument performance (BIP) protocol collects basic information on the hardware, such as the average output power of the pulsed laser source, the repetition rate, the central wavelength, and the width. But, more relevant, BIP prescribes tests on the whole system, which are: (i) the temporal instrument response function (IRF)—its shape, its background, and its stability in time; (ii) the responsivity of the detection system; (iii) the differential nonlinearity (DNL) of the timing electronics.

#### 4.1.1 Instrument response function

Measuring the instrument response function (IRF) is crucial to understand the time resolution of a TD instrument and plays an important role in the model-based reconstruction of the optical properties. The IRF is usually measured by inserting a reference sample in between the source and detector (fibers). The reference sample should be chosen such that it duplicates the measurement conditions (such as filling the acceptance angle of the detectors/detection fibers) without modifying the temporal dispersion. A thin layer of highly scattering materials such as Teflon is typically used for this purpose. A detailed discussion of the IRF and the various factors that influence it can be found in Ref. 18. However, as a first approximation, we consider the full-width at half-maximum (FWHM) measured in picoseconds to be the relevant metric or representative of the IRF. In other words, the FWHM of the IRF (at a specific wavelength) will be used as one of the synthetic descriptors.

#### 4.1.2 Responsivity

The responsivity of the detection system in DO is a measure of the efficiency of detecting low light levels emerging from the tissue. In general, the responsivity of a detector is the ratio between the measured signal and the magnitude of the input illumination. In the present context, it is defined as the ratio of the photons counted by the TD instrument to the photon radiance exiting the diffusive sample. This measurement is performed with a specific “responsivity phantom” [Fig. 2(a)] with known diffuse transmittance factor that acts as an approximately uniform light source with Lambertian angular characteristics.^18^ A transmittance measurement is performed on this phantom and the number of photons collected at the detector over a specified time is recorded. The power input to the phantom at this specific configuration is also measured. Then substituting these values in the following formula gives the responsivity of the detector:

$$s_{\det}^{L}(\lambda )=N_{\mathrm{tot}}/[t_{\mathrm{meas}}\kappa_{p}(\lambda )P_{\text{in}}(\lambda )],$$ (1)

where $\kappa_{p}(\lambda )$ is the phantom-specific photon transmittance factor (in units of $W^{-1}\;s^{-1}\;m^{-2}\;{\mathrm{sr}}^{-1}$), $P_{\text{in}}(\lambda )$ is the input power at the specific wavelength (in W), $N_{\mathrm{tot}}$ is the total counts measured (after background subtraction) over a measurement time $t_{\mathrm{meas}}$. The unit of $s_{\det}^{L}(\lambda )$ is $m^{2}\;\mathrm{sr}$. The responsivity of the instrument will be considered as the synthetic descriptor for this test.

**Fig. 3:**
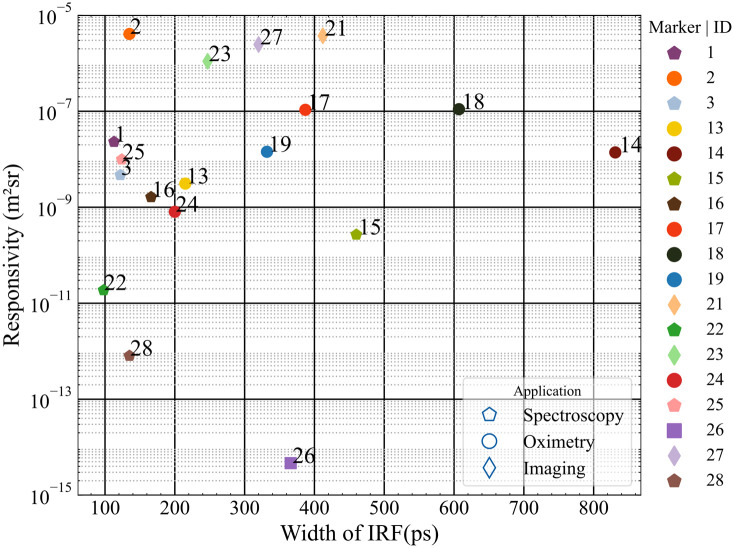
Plot of the responsivity and FWHM of the IRF of TD instruments (λ∼830  nm).

Figure 3 shows the responsivity of the eligible TD instruments against their corresponding FWHM (all values considered at/close to 830 nm). The instrument ID is annotated next to the data point while the application is distinguished by the marker shape in the legend, as for all subsequent population-wide plots. The spread suggests no direct coupling between these two parameters, though some general increase of FWHM upon increasing the responsivity is observed. The relatively large responsivity of instruments #2, #21, #23, and #27 corresponds to the use of large-area SiPM detectors and the two different embodiments of an optical mammograph (Mammot), respectively. All these devices work with the detector directly in contact with the sample (in this case the responsivity phantom). This explains the larger responsivity of overcoming the limitation in numerical aperture and collecting area posed when using optical fibers and bundles. Most of the spectroscopy systems (hexagons) occupy the left-most part of the chart corresponding to shorter FWHMs. This substantiates the fact that the choice of the detector is much dependent on the target application of the instrument.

#### 4.1.3 Differential nonlinearity

Differential nonlinearity (DNL) measures the non-uniformity of the time channel width in a time-correlated single-photon counting (TCSPC) system. It appears as an almost random modulation of the recorded constant photon distribution and can be corrected by a numerical equalization of the width of the time channels in the case of static DNL.

The DNL is recorded as a response to a continuous signal. A battery-powered light source is preferable to avoid any electrical interference. To obtain the DNL with a good signal-to-noise ratio, each time channel should contain $\geq 10^{5}$ counts. Ideally, the photon counts in all-time channels are expected to be equal. The deviation from this situation is characterized by the peak-to-peak difference normalized to the mean value

$$\varepsilon_{\mathrm{DNL}}=\frac{N_{\mathrm{DNL},\max}-N_{\mathrm{DNL},\min}}{\left\langle N_{\mathrm{DNL}}\right\rangle }.$$ (2)

#### 4.1.4 Dark count rate

The dark count rate is another important feature that influences the dynamic range of the instruments’ response in time domain DO. The signal-independent background due to dark counts and residual ambient light can be obtained from a “dark” measurement with the laser source removed.

**Fig. 4:**
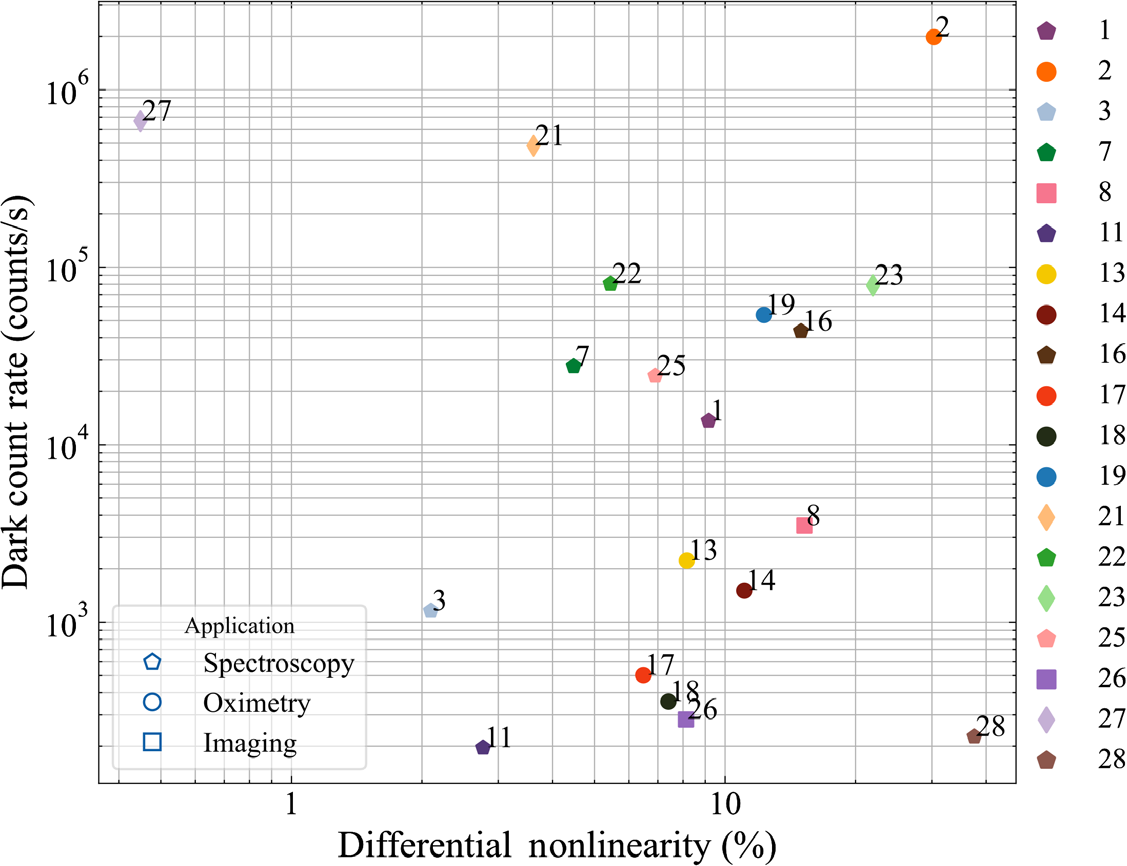
Plot comparing the dark count rate and DNL of TD instruments.

The dark count rate and the DNL as defined in Eq. (2) are plotted in Fig. 4. The instruments which exhibited large responsivity in Fig. 3 (#2, #21, and #27) also demonstrate the highest values of dark count rate. While high dark count rates could reduce the dynamic range of the measurement this loss can be partially recovered by subtracting a common background value of counts.

The DNL does not seem to correlate with any particular category of instruments, being ultimately related to the compromise in cost/complexity of the TCSPC electronics. The range is quite large, but the actual impact on clinical measurements is not necessarily important, otherwise, it can be corrected. For instance, when photons are summed over large (∼500  ps) temporal gates for contrast measurements, the DNL has usually minor effects.

### 4.2 MEDPHOT Protocol

Formulated in the early 2000s, MEDPHOT is a PA protocol designed under the European thematic network with the same name. The different tests outlined in this protocol characterize the instruments’ capabilities to accurately retrieve the absorption and reduced scattering coefficients. For this reason, only instruments capable of recovering absolute optical properties are eligible for this protocol. A detailed explanation of the protocol along with the tests involved can be found here.^19^ Some of the tests in this reference article use “conventionally true” values of the optical properties and compare the results from the experiments to these values. However, in the interest of an unbiased understanding of the results, the same tests when applied here are slightly modified to eliminate the need for such “conventionally true” values.

Some general considerations for all the measurements performed as a part of the MEDPHOT protocol are:

• The standard acquisition time of measurements was 1s.
• Every measurement was repeated 20 times, the results presented are the average of the 20 measurements, and the standard deviation over the 20 measurements is plotted as error bars (wherever applicable).
• Apart from the accuracy and linearity measurements (which were performed over the entire MEDPHOT kit) all the other tests were performed on the B2 Phantom of the MEDPHOT kit (nominal values at 800 nm: $\mu_{a}=0.05\;{\mathrm{cm}}^{-1}$, $\mu_{s}^{\prime }=10\;{\mathrm{cm}}^{-1}$).
• The target count rate from the TD instrumentation was $5\times 10^{5}\;s^{-1}$. But this particular condition was more suggestive than restrictive (in case the standard operating conditions of the instruments demanded a different count rate, as for large-area SiPM detectors with high dark count rate).

#### 4.2.1 Accuracy

**Fig. 5:**
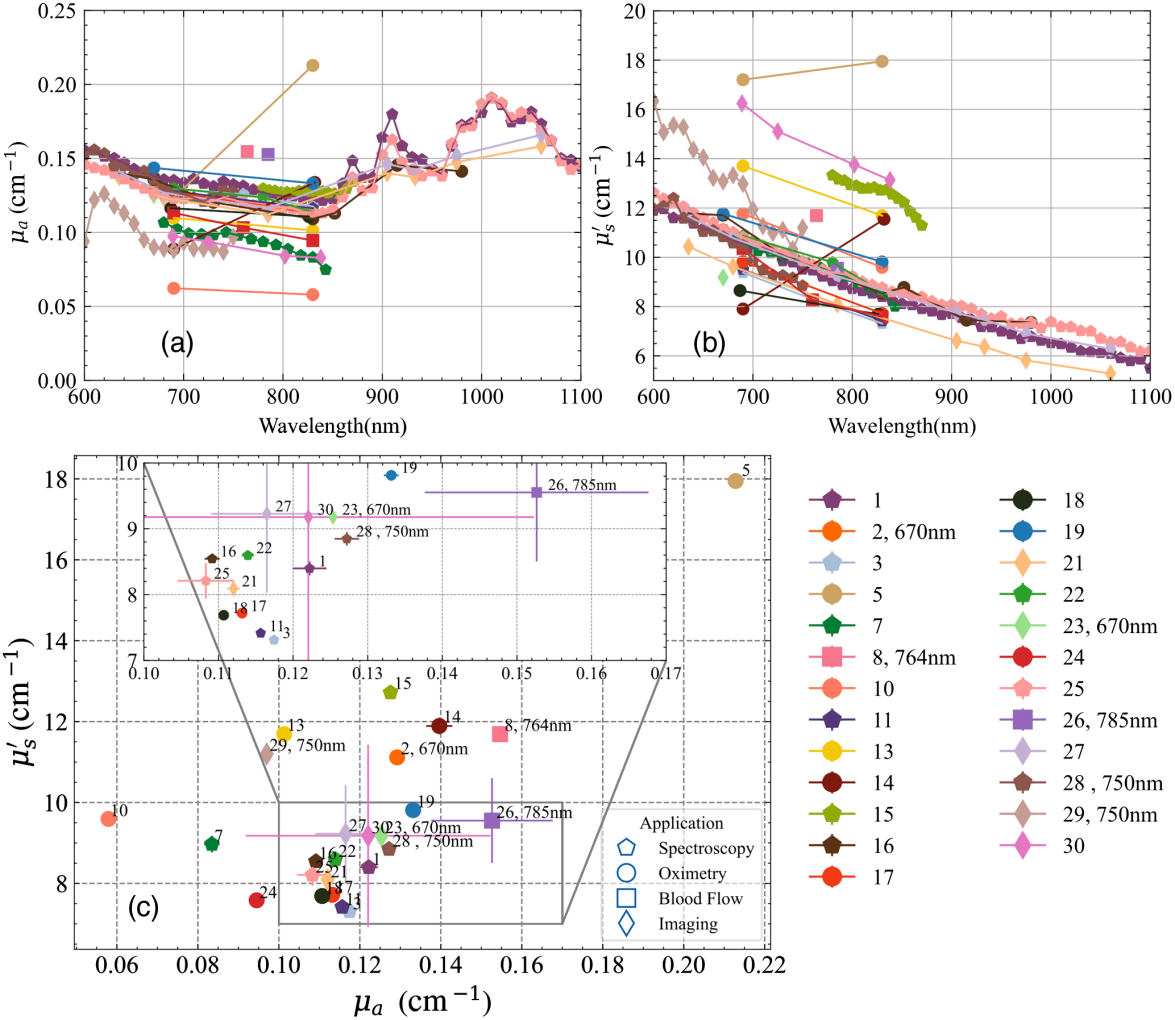
The absorption (a) and reduced scattering and (b) spectra of all the TD instruments measured on the phantom B3 of the MEDPHOT kit. The panel (c) shows these optical properties plotted against each other at 830 nm (wavelength mentioned in cases where it is not 830 nm). Results represent the average over the 20 repetitions with the standard deviation plotted as error bars. The inset box is a zoom on the overlapped data points. The instrument ID is annotated next to the data point and presented as a legend to the right.

The accuracy test addresses the capability of the system to retrieve the absolute estimate of the absorption and reduced scattering coefficients of a reference medium or phantom. As an example, Figs. 5(a) and 5(b) display the absorption and reduced scattering coefficients versus wavelength obtained from all the instruments when measuring one of the phantoms (B3, nominal values at 800 nm: $\mu_{a}=0.1\;{\mathrm{cm}}^{-1}$, $\mu_{s}^{\prime }=10\;{\mathrm{cm}}^{-1}$) of the MEDPHOT kit. Figure 5(c) shows the optical properties provided by different instruments at 830 nm (data for instruments not operational at this wavelength are provided at the wavelength closest to 830 nm). Overall, the median deviation of instruments operating at 830 nm is 9% and 12% of the median value for absorption and reduced scattering, respectively. The data point with the maximum deviation from the rest (#5) corresponds to one of the frequency domain instruments in the cohort, still this discrepancy could be due to the calibration procedure of this device rather than to the technique itself.

#### 4.2.2 Linearity and crosstalk

**Fig. 6:**
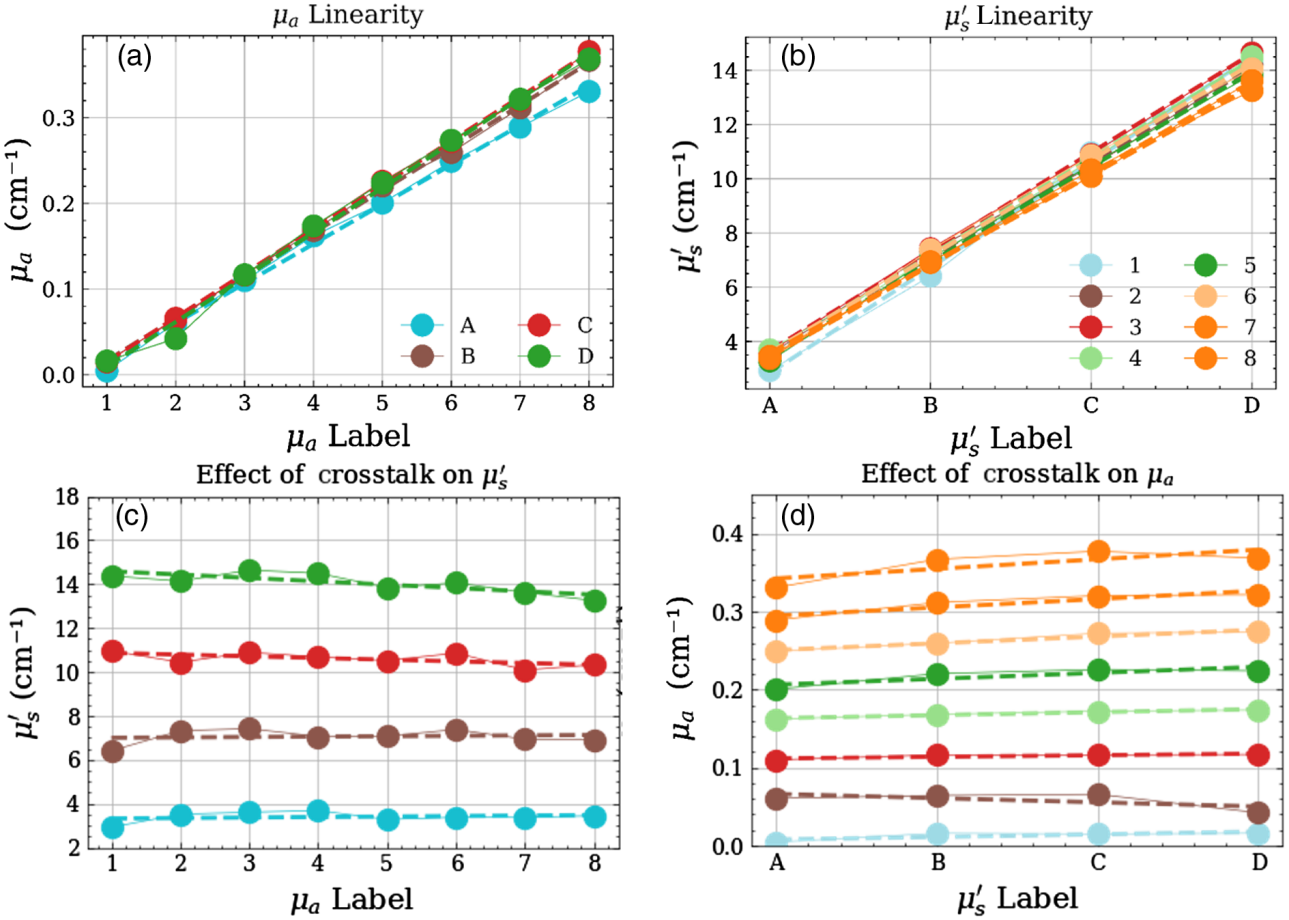
An exemplary plot of the linearity and crosstalk between the optical properties. Panels (a) and (b) show the linear increase in the absorption and reduced scattering coefficients for each series corresponding to their labels (x-axis). Panels (c) and (d) show the influence of one optical property on the other.

The aim of this test is to ascertain the linearity in retrieval of the optical properties which grants—for instance—the preservation of the spectral shape, and also to characterize the unwanted crosstalk between the two optical parameters leading to artefacts in the estimate of optical properties. Figure 6 shows an exemplary plot for a specific instrument (#11). The upper row displays the linearity in $\mu_{a}$ [Fig. 6(a)] and $\mu_{s}^{\prime }$ [Fig. 6(b)], respectively. The lower row represents the absorption-to-scattering [Fig. 6(c)] and scattering-to-absorption [Fig. 6(d)] crosstalk, respectively. Ideally, points should be lying on the regression line in the top row and on horizontal lines in the bottom row, irrespective of the absolute values.

The synthetic indicators are obtained in the following way. For the linearity plots, i.e., Figs. 6(a) and 6(b) the median value of the relative deviation of the data points and the linear fit (dashed line) over the different series is considered to represent the median deviation from linearity for the specific optical property.

For the crosstalk plots in Figs. 6(c) and 6(d), the median value of the absolute slopes of the linear fit (dashed line) over the different series is considered a factor representative of the coupling between the two optical properties. Yet, to provide a more directly intelligible indicator, we prefer to refer to the coupling to a relative, rather than absolute changes in optical properties. In detail, let $S_{\mu_{s}^{\prime }/\mu_{a}}$ be the median of the absolute slopes of the linear regression of the different series in scattering [Fig. 6(c)], and $\Delta \mu_{a}^{\text{cause}}$ be a variation introduced in the absorption coefficient. Then, $\Delta \mu_{s}^{\prime \text{effect}}$ is the corresponding variation introduced in the reduced scattering coefficient due to the inherent coupling between the two parameters, i.e.,

$$\Delta {\mu_{s}^{\prime }}^{\text{effect}}=S_{\mu_{s}^{\prime }/\mu_{a}}*\Delta \mu_{a}^{\text{cause}}.$$ (3)

Now, expressing the same effect in relative terms with respect to reference optical properties ($\mu_{a}^{0}=0.1\;{\mathrm{cm}}^{-1}$, $\mu_{s}^{\prime 0}=10\;{\mathrm{cm}}^{-1}$) we obtain

$$\frac{\Delta \mu_{s}^{\prime \text{effect}}}{\mu_{s}^{\prime 0}}=F_{\mu_{s}^{\prime }\to \mu_{a}}\frac{\Delta \mu_{a}^{\text{cause}}}{\mu_{a}^{0}},$$ (4)

where we define $F_{\mu_{s}^{\prime }\to \mu_{a}}=S_{\mu_{s}^{\prime }/\mu_{a}}\frac{\mu_{a}^{0}}{\mu_{s}^{\prime 0}}$ as the relative absorption-to-scattering coupling coefficient, which will be used as the FOM for crosstalk. A similar definition is provided for the relative scattering-to-absorption relative coupling, i.e., $F_{\mu_{a}\to \mu_{s}^{\prime }}=S_{\mu_{a}\mu_{s}^{\prime }/\mu_{a}\mu_{s}^{\prime }}\frac{\mu_{s}^{\prime 0}}{\mu_{a}^{0}}$. For example, referring to Fig. 6(d), a relative scattering-to-absorption crosstalk $F_{\mu_{s}^{\prime }\to \mu_{a}}=0.13$ means that any factor resulting in an increment of 10% in the reduced scattering coefficient (cause) is expected to alter the measured absorption coefficient by 0.13×10%=1.3% (effect). Surely, this definition is dependent on the choice of the reference optical properties and must be rescaled for different actual properties, but it is more effective than the absolute deviation from linearity to easily interpret the system performances.

Per these definitions, an ideal instrument would have both these values as close to zero as possible (suggesting perfect linearity in assessing the increasing optical property and zero influence of one parameter on the retrieval of the other).

**Fig. 7:**
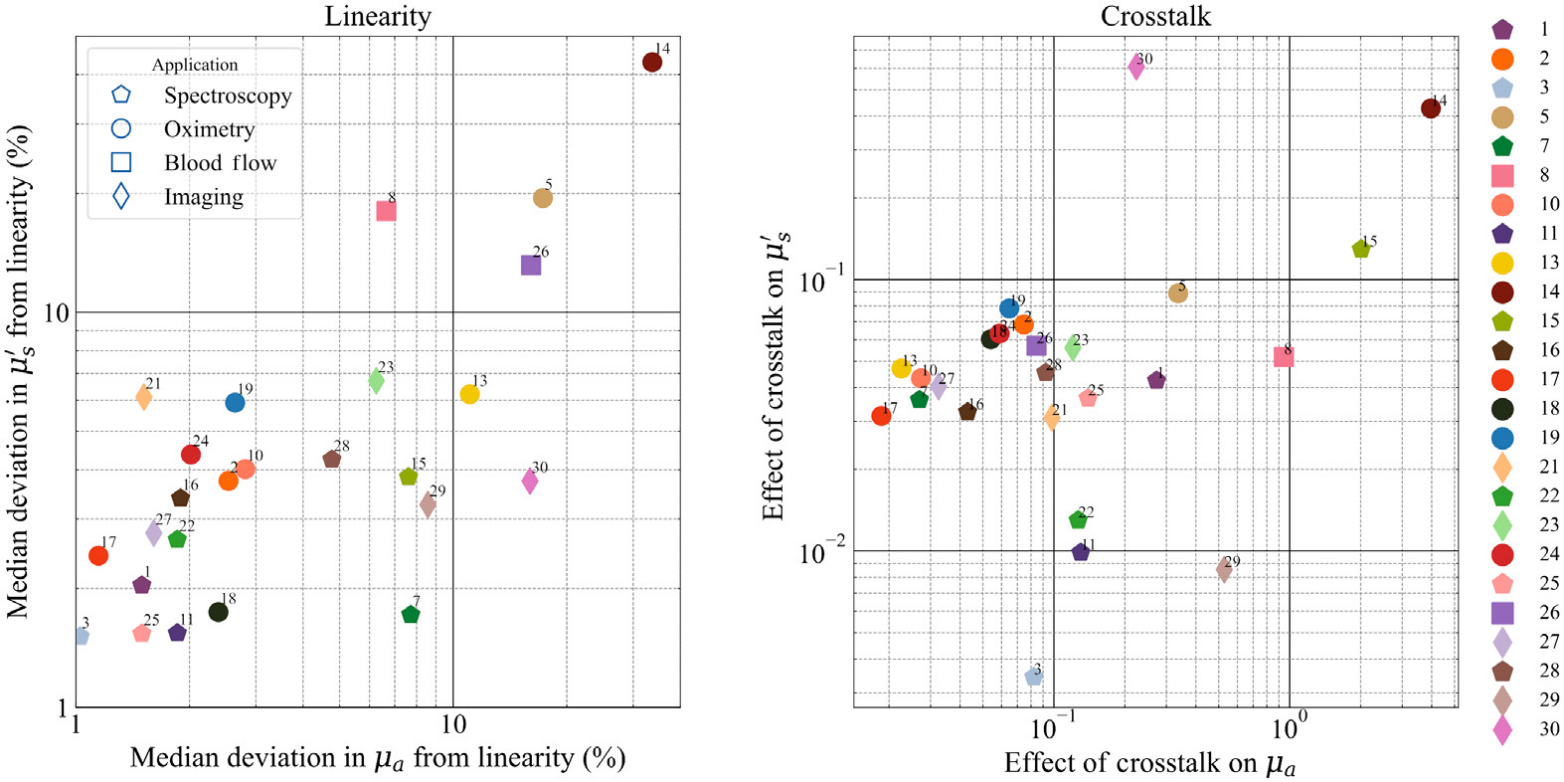
FOM plots for the linearity and crosstalk tests of the MEDPHOT protocol.

Figure 7 presents the resultant plots for the linearity and crosstalk tests for the whole instrument population. The left pane shows the linearity of the two optical properties against each other (absorption on the x-axis and reduced scattering on the y-axis) while the right pane shows the crosstalk for the same properties. In the plot for linearity, 20 out of the 24 instruments enrolled exhibit a median deviation from linearity in both optical properties under or close to 10%. The instruments designed for TD-DCS (blood flow, #8, #26) and the frequency domain instrument (#5) show a little larger deviation in linearity. Again, most of the spectrometers (hexagons) are seen to have a deviation better than 3% in the linearity of reduced scattering and better than 2% in the linearity of absorption. In general, there is a trend of correlation between the deviations in linearity in absorption and in scattering, which is reasonable since systems more optimized for, e.g., spectroscopy is designed to accommodate large variations in signal intensity—by means for instance of low background noise—and variations in the shape of the distribution of time of flight (DTOF)—by adopting a detector with a narrow IRF and high dynamic range.

The plot for crosstalk, shows—for most of the instruments—a relative scattering-to-absorption ($F_{\mu_{s}^{\prime }\to \mu_{a}}$) and absorption-to-scattering ($F_{\mu_{a}\to \mu_{s}^{\prime }}$) crosstalk <20% and <10%, respectively.

#### 4.2.3 Stability

**Fig. 8:**
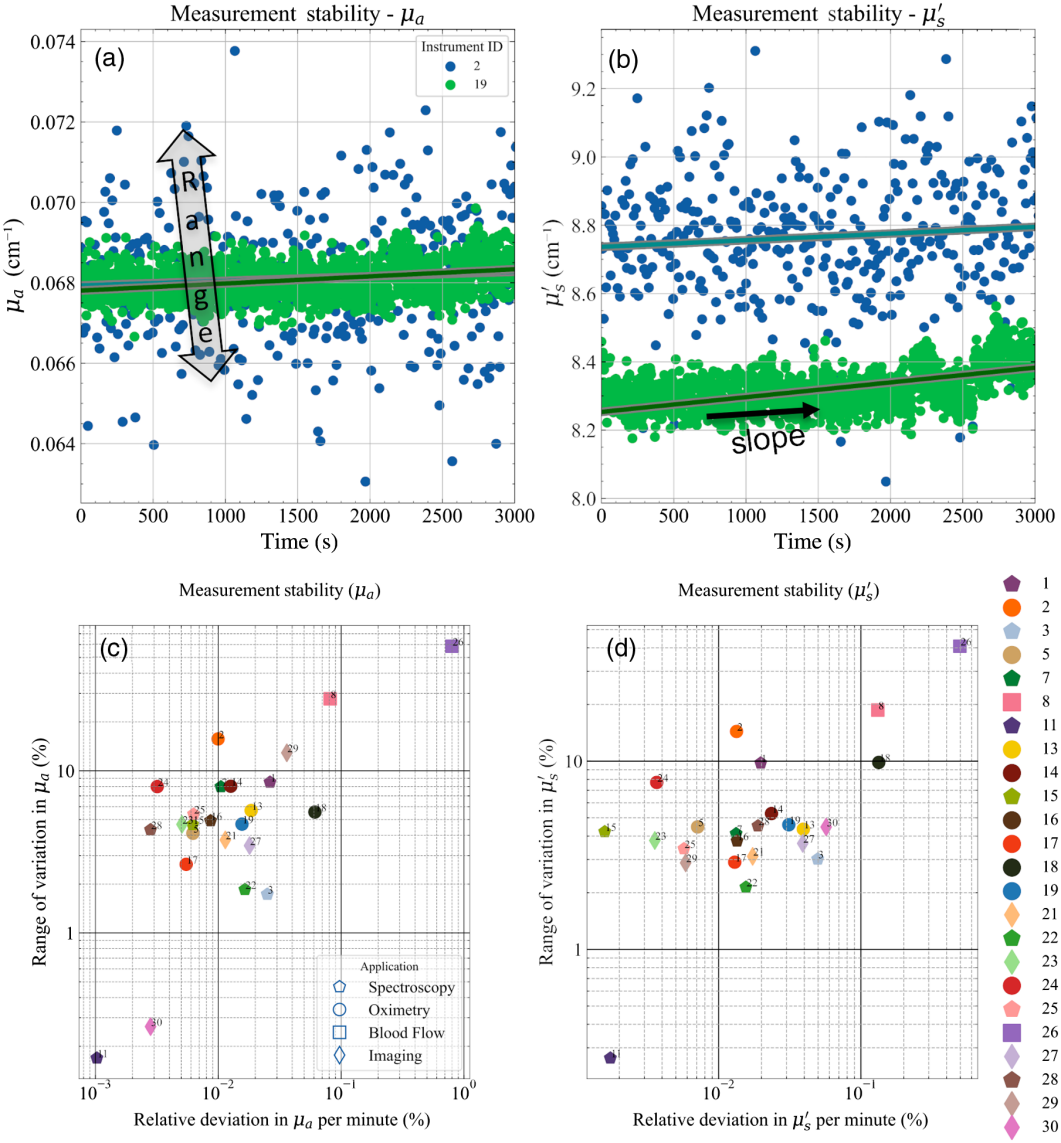
(a), (b) Example of the measurement stability plots for instruments 2 and 19 (on the B2 phantom) with synthetic indicators range and slope depicted. (c), (d) The corresponding FOMs.

Figures 8(a) and 8(b) display some exemplary plots of the temporal stability in the retrieved optical properties based on measurement over a period of >1  h for two instruments. Both the absorption and reduced scattering coefficients are stable within a range of ±10% for #2 while under 4% for #19. In this case, the range of variation over the entire measurement period and the drift given by the slope of the temporal evolution plots were considered as the synthetic indicators.

Figures 8(c) and 8(d) plot the above-mentioned synthetic indicators for both optical properties for all instruments. Most of the instruments lie in the region with a range under 10% for both optical properties. Also, in most cases, the drift (slope) in estimated properties is <0.03% per minute. This means that using any of these instruments for continuous monitoring of the optical properties in a clinical environment, one can expect a maximum deviation of 0.03% in $\mu_{a}$ in 1 minute (or 3% in 100 min).

#### 4.2.4 Noise/uncertainty

**Fig. 9:**
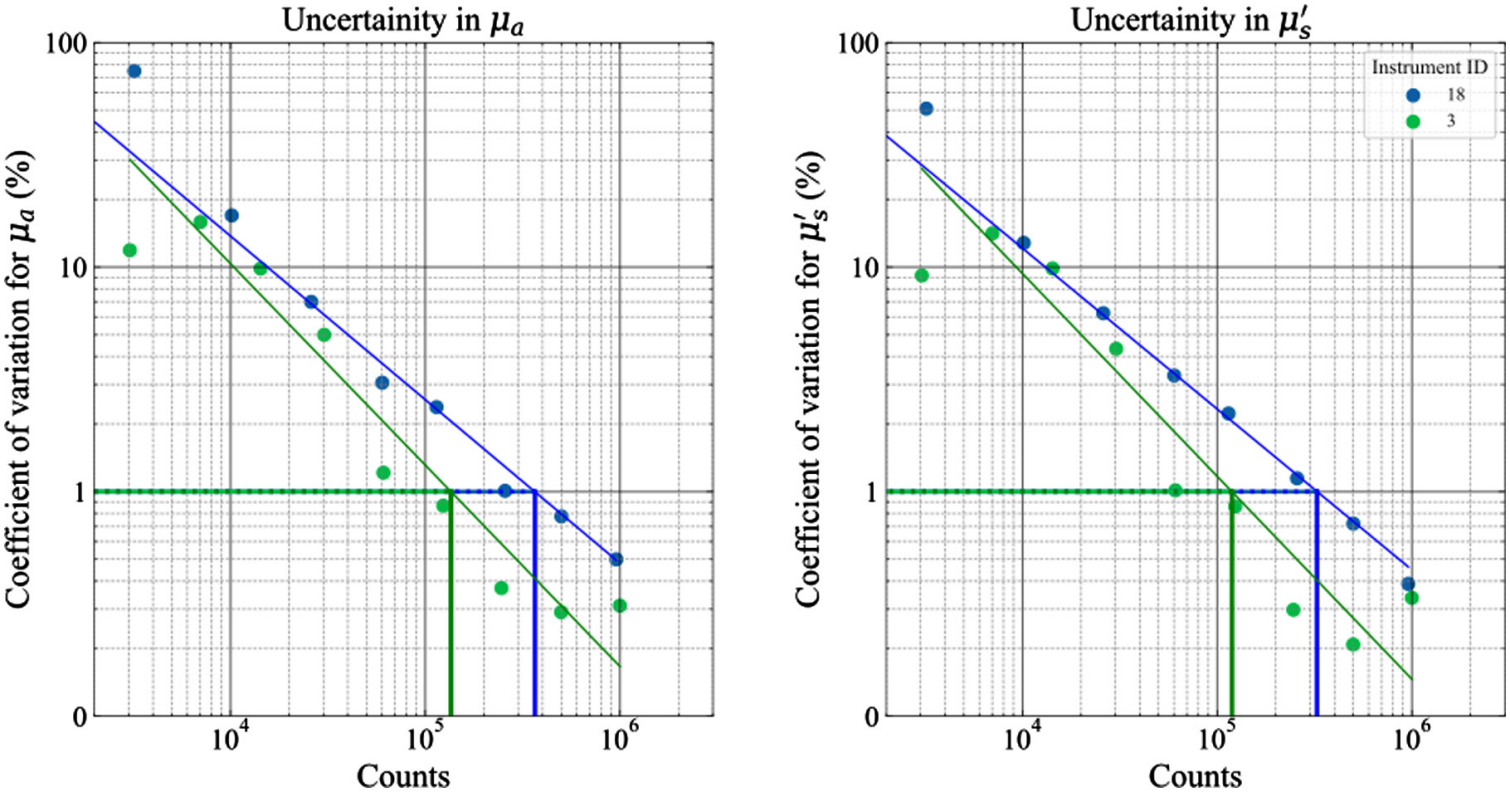
Coefficient of variation (%) in the optical properties plotted against the number of counts for instruments #3 and #18 (test performed on the B2 phantom). A linear fit is performed to determine the number of counts necessary to achieve a CV of 1%. These values are used as the FOMs in Fig. 10.

A test of the influence of the collected energy (or total counts) on the uncertainty of the measured optical properties is performed by measuring the time-of-flight signals at different count rates. About 20 acquisitions, with 1-s acquisition time, were taken at different count rates. The coefficient of variation CV (defined as the ratio of the standard deviation of repetitive measurements over the mean value) for the retrieved optical properties at each count rate is plotted against the total counts as shown in Fig. 9. As a general practice, a CV = 1% can be considered a reasonable target for the uncertainty of DO measurements.

The noise/uncertainty plot identifies the minimum number of counts (related to input energy) required to reach such a goal (the horizontal lines in Fig. 9). This is further dependent on the maximum count rate of the system or the maximum input power and correspondingly affects the acquisition time.

**Fig. 10:**
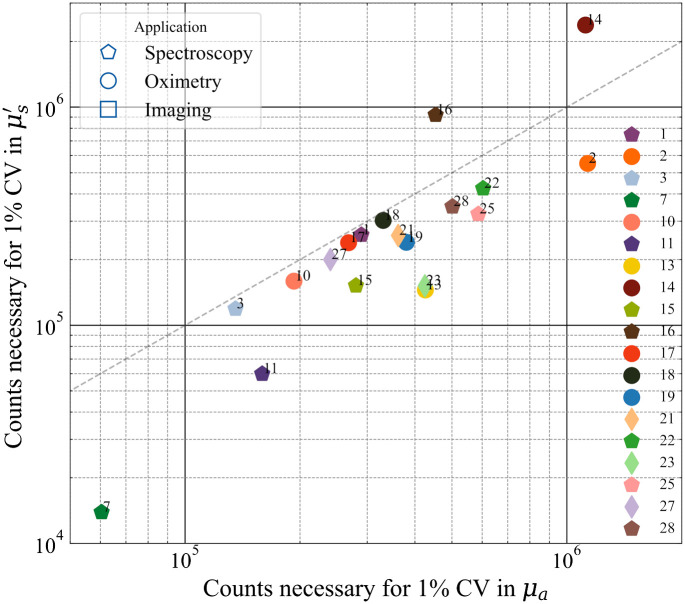
Comparison of the different instruments in terms of the noise/uncertainty measurement.

The synthetic indicators chosen for the noise test are the number of counts necessary to reach a CV of 1% in both the optical properties. Figure 10 plots the counts necessary to achieve 1% CV in $\mu_{s}^{\prime }$ against counts necessary to achieve 1% CV in $\mu_{a}$. The requirement for a good CV in most cases is between $10^{5}$ and $10^{6}$ counts and, in most cases, it is closer to the former. Also, all the results are not far from the line of identity in the plot suggesting the count rate necessary to achieve 1% CV is nearly the same in both optical properties. An interesting observation in this regard is that instrument #7 relies on the method of moments for fitting requires a substantially lower number of counts to achieve a minimal variation in the results as compared to the rest of the instruments. Thus, it would be interesting to understand how the usage of this method of analysis (which is different from the traditional analytical solution based on the DE employed for a majority of the other instruments enlisted) fares with the other instruments. These kinds of studies will be undertaken in Action3 mentioned above.

#### 4.2.5 Reproducibility

**Fig. 11:**
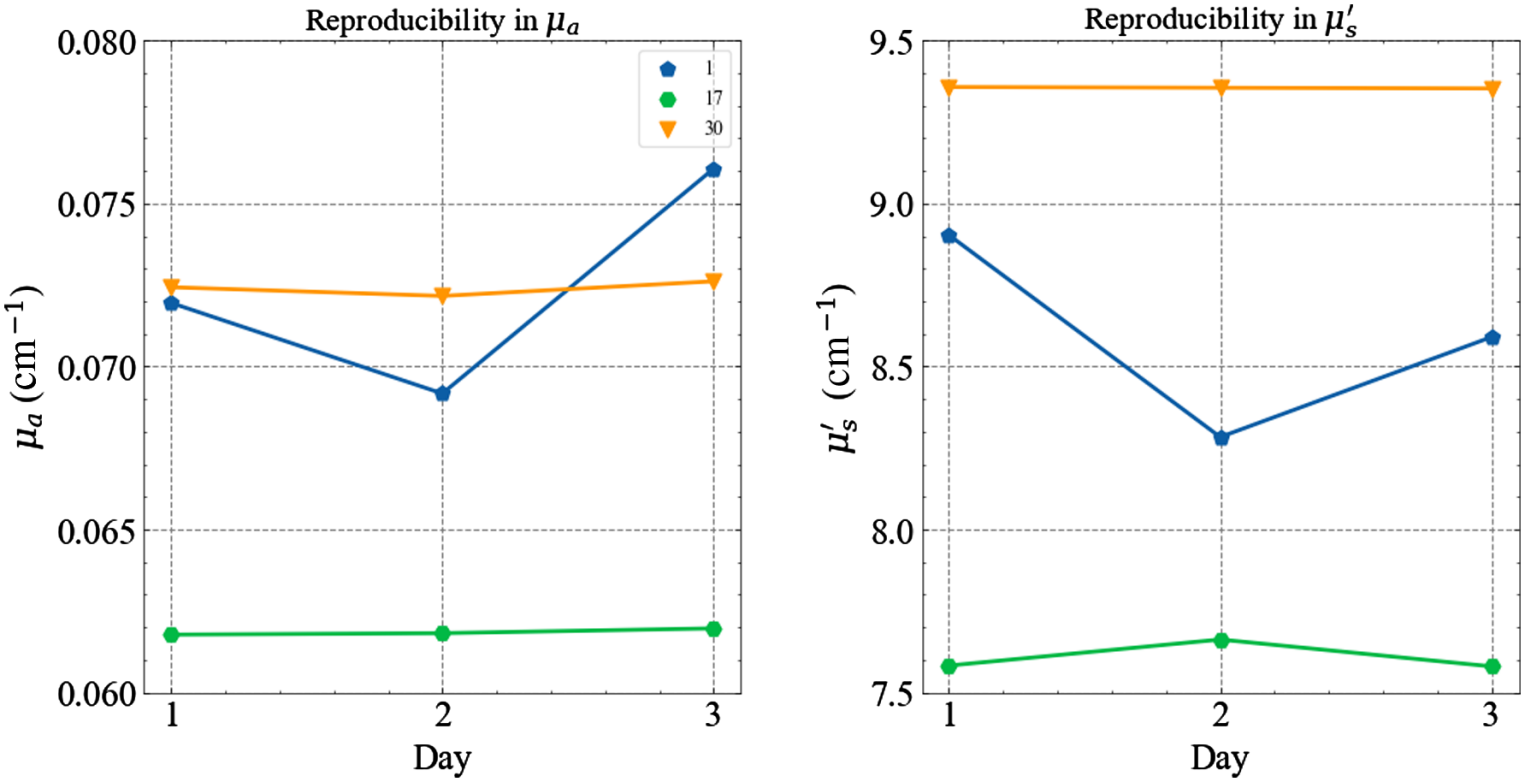
Day-to-day reproducibility in both the optical properties for some of the instruments at 830 nm (all measured on phantom B2 of the MEDPHOT series).

The reproducibility test, as the name suggests, is a general test of how reproducible the instrument’s performance is on a day-by-day basis. Figure 11 displays the reproducibility of three instruments. Data were taken over three different measurement sessions (usually spanning three different days).

**Fig. 12:**
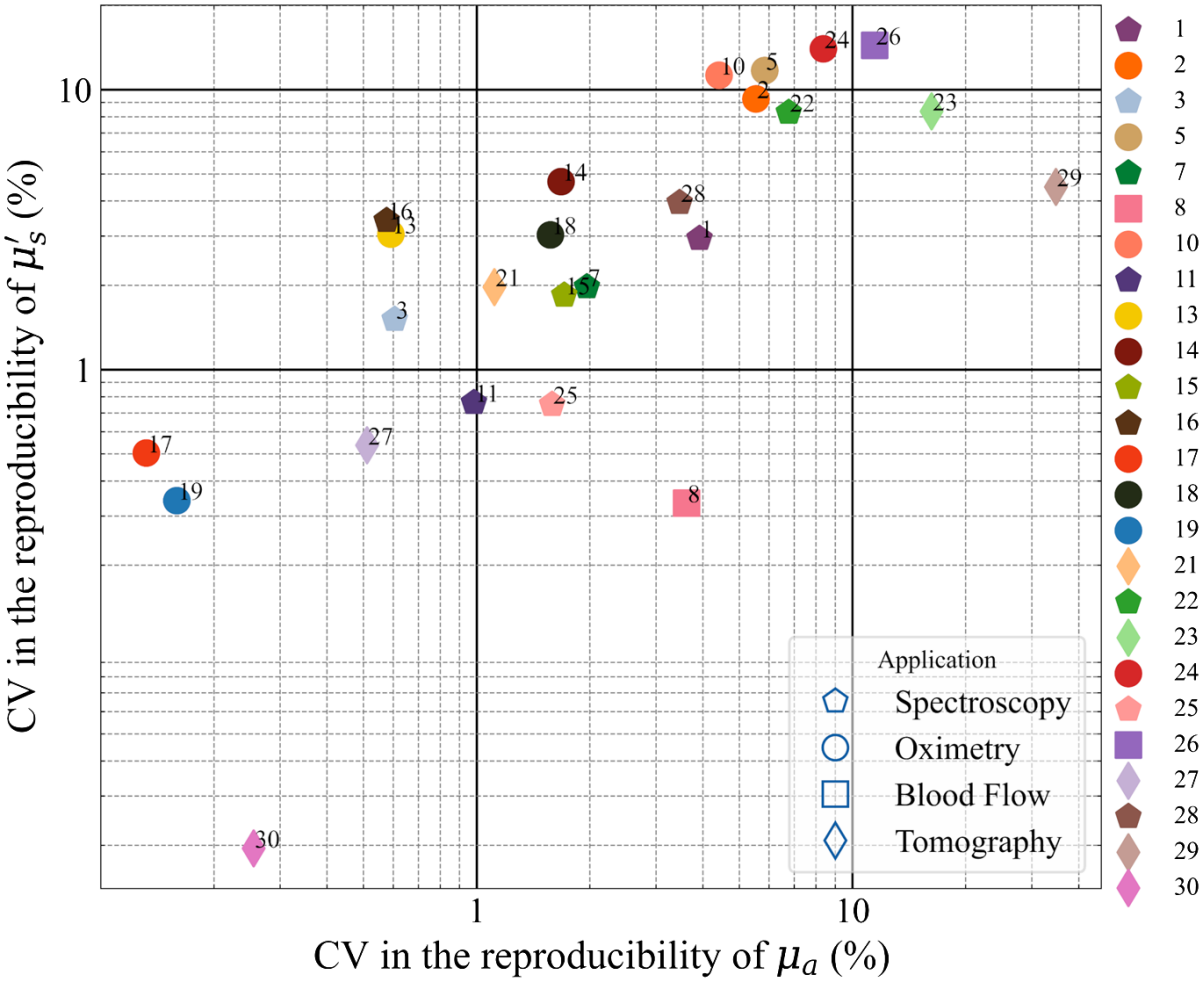
Comparison between instruments for the day-to-day reproducibility expressed as a CV.

As a synthetic indicator for this case, we adopted the CV over the three measurement sessions, which is plotted for the whole population in Fig. 12 for both optical properties.

Generally (>70% of instruments), the reproducibility is better than 5% in both optical properties with some of them better than even 1%. Such testing is critical in a clinical scenario and in general, represents a good scientific conduct. Instruments with relatively large values of CV can still be utilized as long as sufficient measures are taken to address this concern. A good example of this would be the commercial frequency-domain instrument enrolled in this study (#5). A phantom (provided by the manufacturer) with known optical properties is generally used to calibrate the instrument before clinical use, which improves the reproducibility in the results.

### 4.3 nEUROPt Protocol

While it was originally developed and first applied in the context of time-domain optical brain imaging, this protocol can be applied to other modalities as well, such as continuous wave and frequency domain. Two of the tests from this protocol were chosen for the BitMap exercise, namely the Contrast and Lateral Resolution tests. Out of these, we present here the results from the contrast measurements.

#### 4.3.1 Detection of an inhomogeneity: contrast and contrast-to-noise ratio

To ascertain the depth sensitivity of instruments to localized optical perturbations—e.g., functional imaging of brain activity—the systems were tested on an inhomogeneous phantom made of a bulk homogeneous material holding a rod with an embedded inclusion [Fig. 2(c)]. A detailed description of the test can be found in Ref. 20. Briefly, the test involved measuring the DTOF signals (for TD) or photocurrent (for CW) on the phantom seen in Fig. 2(c) in reflectance with the inhomogeneity moving deeper into the phantom. This depth scan is realized by placing the optodes on the side surface of the phantom [at the positions marked in Fig. 2(c)].

Then, the contrast is defined as the relative difference in total photon counts given as

$$C_{i}=(M_{i}-M_{0})/M_{0},$$ (5)

where $C_{i}$ is the contrast at position i, $M_{i}$ corresponds to the number of counts in a certain time window with the inclusion at position i and $M_{0}$ is the corresponding number of counts on the DTOF measured on a homogeneous region (far from the inclusion) of the phantom.

Since each measurement was repeated for 20 times, this also allowed for a calculation of the contrast-to-noise ratio (CNR) given as

$${\mathrm{CNR}}_{i}=(M_{i}-M_{0})/\sigma (M_{0}),$$ (6)

where $\sigma (M_{0})$ refers to the standard deviation of the 20 acquisitions performed at each position at the baseline/homogeneous state.

The black inclusion used for this exercise has a diameter of 0.5 cm and a length of 0.5 cm. The equivalent perturbation/inhomogeneity in absorption ($\Delta \mu_{a}$) achieved by this inclusion is $0.17\;{\mathrm{cm}}^{-1}$ supposing an effective volume of $1\;{\mathrm{cm}}^{3}$ and a background $\mu_{s}^{\prime }$ of $10\;{\mathrm{cm}}^{-1}$.^29^

The two parameters described above, namely the contrast and the CNR ratio will be used as the synthetic indicators for this test. For time-domain instrumentation, the resultant DTOFs can be sliced in time and the counts from the resultant “time-windows” can be inserted in the Eqs. (5) and (6) to get the contrast and CNR at specific time windows. The DTOFs measured in the BitMap exercise were divided into time windows of 400 ps width which were then used to plot the contrast at early and late windows.

**Fig. 13:**
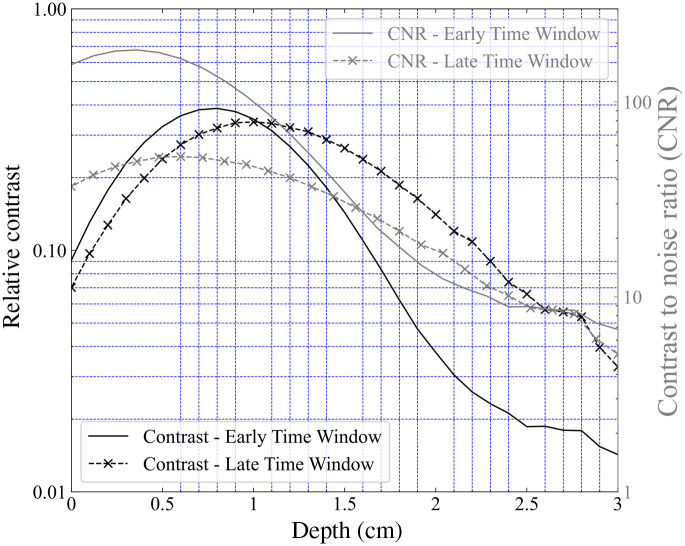
Depth dependent contrast and CNR values plotted against the inclusion depth for the Z-scan for an early and late time window (Instrument #16).

Exemplary plots of contrast and CNR for the depth scan at an “early” (corresponding to the time interval 400 to 800 ps) and “late” time window (corresponding to the time interval 2000 to 2400 ps) for instrument #16 can be found in Fig. 13. The contrast plots at early and late windows suggest that for early time windows the peak contrast is observed at shallower depths (at around 7 mm in this case) while the late windows see maximum contrast at deeper regions (around 11 mm). The CNR values as a function of depth have profiles similar to the contrast profiles. The maximum value of CNR at the early window is, however, much higher than the maximum value at a late window (logarithmic axis).

**Fig. 14:**
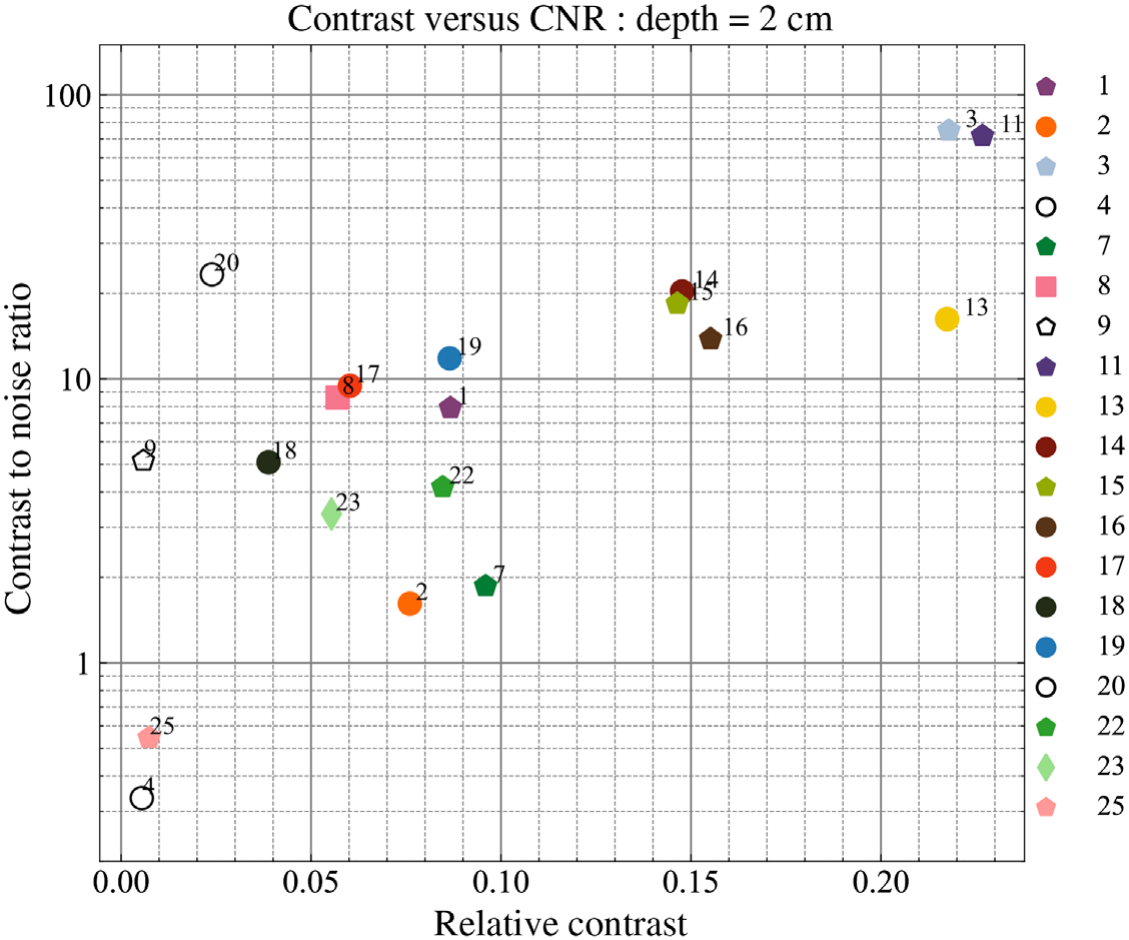
Figure of merit plot for the contrast test of the nEUROPt protocol (contrast versus CNR at an inclusion depth of 20 mm).

The contrast and CNR values at the late window at a depth of 20 mm were chosen as the two synthetic indicators for this particular test. Since the concept of windowing is applicable only to the TD instruments the contrast and CNR values of the CW instruments were calculated based on the total measured counts. The resultant plot is shown in Fig. 14.

A good spread is evident both in contrast and CNR values over all the instruments enrolled. Literature suggests that the depth-dependent contrast when analyzing time windows is influenced by the IRF.^20^ This is confirmed from the results since all the instruments which have a hybrid PMT or MCP-based detection system (i.e., # 11, 3, 13, 14, and 15) are clustered at the top right corner of the plot suggestive of better contrast and CNR values. This could be attributed to the IRF profiles of these instruments which have a fast-decaying tail with almost negligible influence at later photon arrival times. On the contrary, silicon-based detectors have an exponentially decaying tail which could affect the performance of the instruments employing these detectors (# 1, 2, 22, 23, 25, and 19) thus leading to relatively lower values of contrast. Similarly, higher values of CNR were observed for instruments with higher responsivity since this implies lower photon noise for the same acquisition time and interfiber distance. CW instruments (empty markers) show very low values of contrast suggesting poor sensitivity at large depths (20 mm). The improved depth sensitivity for TD systems is due to the increasing mean photon depth upon increasing photon traveling time resulting in higher depth sensitivity for late time windows.^63^

## 5 Discussion

Table 4 summarizes the key statistical descriptors of the synthetic FOMs presented above in the summary figures. The table reports the number of instruments tested for each FOM (counts), the minimum, maximum, mean, and standard deviation of the distribution, and the inferred values corresponding to the 25%, 50% (median), and 75% percentiles. Starting from the BIP protocol, and specifically from the FWHM of the IRF, applicable only to the TD system, sub-ns performances are always retrieved with typical values in the 150 to 400 ps range (25% to 75% percentile). The responsivity spans almost nine decades, encompassing systems equipped with single-mode fiber (DCS) or very large area detectors, with a median value of $10^{-2}\;{\mathrm{mm}}^{2}\;\mathrm{sr}$. Large differences in dark count rate and DNL are observed, spanning a range of 4 and 2 orders of magnitude (200 to 2,000,000  counts/s and 0.4% to 40%) with median values of 10,000  counts/s and 8%, respectively. These huge differences reflect the wide heterogeneity of instruments, encompassing various photonics devices. These FOMs can be further studied to investigate the impact of hardware performances on clinically related results.

**Table 4:** Summary statistics of the synthetic FOMs.

Protocol	Test	Unit	FOM	Opt	count	mean	std	min	25%	50%	75%	max
BIP	IRF	ps	FWHM	All	18	293	197	98	135	231	382	831
BIP	Responsivity	mm2sr	Responsivity	All	18	$6.5\times 10^{-1}$	$1.3\times 10^{0}$	$4.7\times 10^{-9}$	$1.0\times 10^{-3}$	$1.2\times 10^{-2}$	$1.1\times 10^{-1}$	$4.1\times 10^{0}$
BIP	DarkCounts	counts/s	Dark counts	All	19	$1.82\times 10^{5}$	$4.71\times 10^{5}$	$1.96\times 10^{2}$	$8.30\times 10^{2}$	$1.36\times 10^{4}$	$6.64\times 10^{4}$	$1.98\times 10^{6}$
BIP	DNL	—	DNL	All	19	11.0%	9.7%	0.4%	5.0%	8.1%	13.6%	37.6%
MEDPHOT	Accuracy	—	Deviation	Mua	25	15.3%	18.6%	0.0%	4.8%	9.2%	18.9%	82.7%
MEDPHOT	Accuracy	—	Deviation	Mus	25	16.7%	19.5%	0.0%	4.5%	11.8%	21.1%	95.5%
MEDPHOT	Linearity	—	Linearity	Mua	25	6.5%	7.6%	1.0%	1.9%	2.6%	7.7%	33.7%
MEDPHOT	Linearity	—	Linearity	Mus	25	6.7%	8.9%	1.5%	2.4%	3.7%	6.1%	43.0%
MEDPHOT	Linearity	—	Crosstalk	Mua	25	38.3%	85.8%	1.9%	5.4%	9.2%	22.4%	397.0%
MEDPHOT	Linearity	—	Crosstalk	Mus	25	8.4%	13.6%	0.3%	3.3%	4.5%	6.3%	60.8%
MEDPHOT	Noise	counts	Counts1%	Mua	19	4.18E+5	2.89E+5	6.05E+4	2.54E+5	3.61E+5	4.77E+5	1.13E+6
MEDPHOT	Noise	counts	Counts1%	Mus	19	3.81E+5	5.24E+5	1.39E+4	1.52E+5	2.40E+5	3.37E+5	2.38E+6
MEDPHOT	Stability	$\min^{-1}$	Drift	Mua	24	0.05%	0.16%	0.00%	0.01%	0.01%	0.02%	0.80%
MEDPHOT	Stability	$\min^{-1}$	Drift	Mus	24	0.05%	0.10%	0.00%	0.01%	0.02%	0.04%	0.50%
MEDPHOT	Stability	—	Range	Mua	24	8.6%	12.1%	0.2%	3.7%	4.8%	8.0%	58.6%
MEDPHOT	Stability	—	Range	Mus	24	6.9%	8.3%	0.3%	3.3%	4.3%	5.9%	40.8%
MEDPHOT	Reproducibility	—	Reproducibility	Mua	25	4.7%	7.4%	0.1%	0.6%	1.7%	5.5%	34.8%
MEDPHOT	Reproducibility	—	Reproducibility	Mus	25	4.5%	4.5%	0.0%	0.8%	3.0%	8.3%	14.4%
nEUROPt	Detection	—	CNR	All	19	20.8	30.7	0.3	3.7	12.5	20.9	104.9
nEUROPt	Detection	—	Contrast	All	19	8.6%	6.5%	0.5%	3.1%	8.4%	14.3%	20.0%

Moving to the MEDPHOT protocol and starting from the accuracy test, to avoid bias due to erroneous knowledge of true optical properties, we describe the accuracy in terms of deviation around the median value. This figure has no meaning for the single instrument because the median is not a substitute for the true value, but it is relevant to describe the disagreement within the whole population. We obtained a median relative deviation of <9% for $\mu_{a}$ and <12% for $\mu_{s}^{\prime }$, with still 75% of instruments within a 20% displacement on both optical properties. In terms of linearity, most instruments perform well (median <4%, 75th percentile <8%). Median crosstalk is around 9% for $F_{\mu_{s}^{\prime }\to \mu_{a}}$ and 5% for $F_{\mu_{a}\to \mu_{s}^{\prime }}$. This means that, e.g., a change of 10% in $\mu_{s}^{\prime }$ yields an artificial increase of roughly 1% in the measured $\mu_{a}$. In terms of noise, for half of the systems $<3.6\times 10^{5}$ and $<2.4\times 10^{5}$ counts are needed to obtain an uncertainty of 1% on $\mu_{a}$ and $\mu_{s}^{\prime }$, respectively. The stability of systems is rather good with a median range of variation of <5% and a median drift of <0.01% per minute. Day-by-day reproducibility on $\mu_{a}$ and $\mu_{s}^{\prime }$ is on the order of <2% and <3%, respectively, for half of the systems and still with an acceptable <6% and <8% for the 75th percentile.

Finally, the nEUROPt protocol addresses the detection of a reference optical inhomogeneity at 2-cm depth within an otherwise homogeneous medium. For time-domain systems, this test depends on the selected time window, and we opted to compare all systems for a 2000- to 2400-ps window. This leads to a median contrast of 9% and a median CNR of 13. We stress again here that these synthetic indicators are obtained for reference conditions (in most of the cases for a background medium with $\mu_{a}=0.1\;{\mathrm{cm}}^{-1}$ and $\mu_{s}^{\prime }=10\;{\mathrm{cm}}^{-1}$) and therefore should be interpreted properly for the real clinical situation.

This large-scale BitMap campaign allowed us to identify some critical issues related to PAS in DO, which we will discuss in the following.

### 5.1 Accurate Multi-Laboratory Characterization of Solid Phantoms

While for liquid phantoms a good level of reliability in optical characterization was reached through multi-laboratory studies,^21^ conversely solid phantoms—which are definitely more suitable for practical use—are still prone to a larger uncertainty in the determination of optical properties. The present BitMap exercise cannot help in this direction since the goal was to compare instruments and not to accurately characterize phantoms. Therefore, the data in Fig. 5 cannot provide an estimate of the “conventionally true” phantom optical properties. What is needed instead—similarly to the process that led to the characterization of aqueous solutions of Intralipid and ink^21^—is a first set of individual works identifying the most suitable characterization approaches, followed by multi-laboratory undertakings to converge to common values. This activity could surpass the specific realm of DO, since it is a common need for many other optical techniques (e.g., photoacoustics, fluorescence, optical coherence tomography, DCS, and diffuse Raman spectroscopy).

### 5.2 Easily Available Common Phantom Kits

The whole BitMap exercise was run using a unique collection of three phantom kits. In the ideal case, the availability of identical or highly reproducible phantom kits easily accessible for any laboratory would permit to repeat the test over time and benchmark system upgrade or development of novel instruments in an absolute way. These tools are already available in other more mature clinical techniques such as MRI and ultrasound. Surely, the above-mentioned issue #1 is a prerequisite.

### 5.3 Reduce the Discrepancy in the Measured Optical Properties

Figure 5 displays a certain level of disagreement among the tested instruments in the recovery of the absolute value of the absorption and reduced scattering coefficients. Accuracy is not necessarily the most critical parameter when dealing with clinical applications, where possibly linearity (MEDPHOT) or detection sensitivity (NEUROPT) could play a major role in the clinical application. Yet, understanding the causes and reducing the discrepancies is an important goal for the next few years. Possible paths to reduce the variation are (i) common analysis tools (see issue #5) with shared guidelines to exclude operator influence together with easily available rigorous models (e.g., through MC fit); (ii) standard reference and well-characterized phantoms (see issue #1) for instruments relying on calibration; (iii) common guidelines or good practices for performing the measurements (e.g., ways to acquire the IRF); (iv) correlation of the discrepancies with specific techniques/technical solutions (the open data to be deployed in Action2 could be further investigated in future). Surely, some discrepancies are unavoidable and intrinsic in the limitations of particular instruments tailored to optimize other requirements rather than accuracy. In any case, multi-laboratory initiatives are mostly needed since single-laboratory efforts could be self-referential and biased on the specific laboratory habits.

### 5.4 Link FOMs to Specific Clinical Features

The three protocols and related FOMs were designed starting from paradigmatic clinical problems. To derive clinical implications from the lab system performances we need to quantify the impact of a given FOM on specific clinical applications. For instance, using a set of equivalence classes, optical perturbations caused by brain activation or breast lesion were quantified in terms of an equivalent black volume (EBV)^29^ which is then directly mapped to the contrast or CNR. For instance, in an exemplary case, a malignant breast lesion was graded at $\mathrm{EBV}\approx 100\;{\mathrm{mm}}^{3}$, while a subtle motor task brain activation at $\mathrm{EBV}\approx 10\;{\mathrm{mm}}^{3}$. Data in Table 4 were obtained for $\mathrm{EBV}\approx 170\;{\mathrm{mm}}^{3}$. Existing clinical datasets could be reanalyzed to link existing FOMs to clinical features and study the impact of system performances on *in vivo* measurements. Surely, the increasing availability of open data sets could unleash meta-analysis of different datasets, although informative metadata is often needed and not standardized yet to interpret DO data.

### 5.5 Analysis

Despite other direct imaging modalities (e.g., X-ray), DO results strongly depend on the model and data analysis in use. Often, it is not easy to disentangle inaccuracies related to the hardware from misfit in the model. The fairly large variability observed in Fig. 6 could be reduced by adopting the very same analysis tool. In this first Action1 we opted to present the results following the analysis approach chosen by each group in daily applications. This should roughly correspond to the expected behavior under clinical applications. In Action3, we will pursue the common analysis of the whole dataset using the very same tools, hoping to reduce variability and identify the most effective and robust analysis methods. We observe a plethora of proposed approaches and implementations in retrieving optical properties of homogeneous media, ranging from the diffusion equation to different orders of approximation of the radiative transport equation, from the random walk to MC tools. Still, proprietary analysis tools, or complex-to-implement analytical solutions hinder reaching consensus or common daily use. Emerging of open software suites is definitely a plus in this direction, and again we need more and more interlaboratory studies or common analysis of multiple datasets.

### 5.6 Interoperable Data Format

Given the enormous effort involved in clinical studies, the possibility to reanalyze existing datasets is of great interest and efficiency. Even consolidated phantom measurements can be used to test new approaches. The adoption of open-source analysis platforms (e.g., HOMER^64^ and NIRFAST^65^) can speed the analysis process and consistency of results. Also, the deployment of open data sets, required by many funding agencies, will offer a wealth of *in vivo* and phantom data. Some attempts in setting data formats for DO were proposed following, e.g., the HOMER^64^ or SNIRF^27^ standards. For the deployment of BitMap open data, we will pursue the latter, proposed by the Society for functional Near-Infrared Spectroscopy.^66^ Although tailored to a specific application, and lacking a bit of generality, yet the SNIRF format can reduce the Babel of individual data specifications to a single data format which then can be easily uploaded to analysis tools or converted to specific formats. Other fields reached impressive results in this respect—e.g., the DICOM format for clinical images—but also emerging areas such as photoacoustics are setting a sound ground through the International Photoacoustic Standardisation Consortium.^67^^,^^68^

## 6 Conclusion

We have presented the largest interlaboratory comparison of PA of DO instruments, enrolling 28 systems and involving >50 researchers out of 12 institutions. The exercise capitalized on two decades of research in the EU leading to three protocols (BIP, MEDPHOT, and NEUROPT) and a set of solid phantoms implementing them. Instruments were based on different techniques, mostly ascribed to time-domain approaches, but encompassing also CW and frequency-domain, finalized for different applications, ranging from oximetry to tissue spectroscopy, from optical mammography to diffuse correlation spectroscopy. The tests assessed different features, mostly ascribed to specific clinical oriented needs, such as accuracy and linearity in the assessment of optical properties inhomogeneous media, the stability of measured values over continuous measurements, and their reproducibility on different days, the sensitivity in detecting optical inhomogeneities buried in-depth in the medium. A large amount of heterogeneous data was generated by the exercise, and we tried to present them in a similar format. Further, we proposed a comprehensive synthetic-summary analysis of the multiple tests based on a set of 20 FOMs, mostly consolidated from previous papers and partially introduced here anew. In Table 4, we provided descriptive statistics of the FOMs for the whole instrument population which could be used as a reference table to benchmark an instrument or simulate applications.

In this study, we identified five needs/criticalities which are (i) the lack of reliable multicenter results on the characterization of solid phantoms; (ii) the need for identical/reproducible phantom kits easily available for research centers; (iii) the benefit of linking physical FOMs to specific features in the clinical measurements; (iv) the role of data analysis and common analysis tools; (v) the demand for standardized formats for open data and data sharing.

Our immediate future actions foresee deployment of the whole dataset in an open data repository with addition of relevant metadata to be able to further analyze specific aspects, such as the influence of the basic instrument performances on the characterization of homogeneous or inhomogeneous media, the role of specific detectors or lasers, and the impact of analysis methods. In particular, as a third action of the BitMap exercise, we foresee to reanalyze the whole dataset using the very same tools to understand to which extent the observed interinstrument variability can be attributed to different analysis methods.

Great advances in physics derived from precise measurements of specific physical quantities (e.g., planet orbits, speed of light, and particle masses). Photon migration through the human body is complicated by the biological variability, but not the basic physics underlying it all. We can disentangle the uncertainties and artifacts produced by the instruments and analysis tools from the biological variability, with great impact on clinical use.

## Data Availability

The data presented in this article will be available on an open data repository soon
